# Development of mental health problems - a follow-up study of unaccompanied refugee minors

**DOI:** 10.1186/1753-2000-8-29

**Published:** 2014-11-17

**Authors:** Tine K Jensen, Envor M Bjørgo Skårdalsmo, Krister W Fjermestad

**Affiliations:** Department of Psychology, University of Oslo, Forskningsveien 3a, 0372 Oslo, Norway; Norwegian Centre for Violence and Traumatic Stress Studies, Gullhaugveien 1-3, 0484 Oslo, Norway; Frambu center for rare disorders, Sandbakkveien 18, 1404 Siggerud, Norway

**Keywords:** Unaccompanied, Refugee children, Mental health, Trauma, Longitudinal

## Abstract

**Background:**

Studies have shown that unaccompanied refugee children have elevated symptoms of post-traumatic stress disorder (PTSD), depression, anxiety, and externalizing problems. Few studies have examined change in this group’s mental health symptoms after resettlement in a new country, particularly for those who arrive to a host country when being under the age of 15.

**Method:**

The sample included 75 unaccompanied refugee children (mean age 16.5 years, SD =1.6; 83% boys) who settled in Norway. We examined change in the number of stressful life events, symptoms of PTSD (Child PTSD Symptom Scale; CPSS), and symptoms of anxiety, depression and externalizing problems (Hopkins Symptom Checklist; HSCL-37A) from 6 months after arrival (T1) to 1.9 years (SD =0.6) later (T2) using paired samples t-tests. Linear regression models were used to examine whether length of stay, level of education or change in the number of experienced stressful life events predicted symptom change.

**Results:**

There was a small and non-significant change in the mean scores of both symptom scales between T1 and T2, although there was considerable variation among the participants. The number of children who remained above the clinical cut-off value from T1 to T2 was as follows: 28 of 47 (59.6%) on the CPSS and eight of 16 (50.0%) on the HSCL-37A. There was a significant increase in the number of reported stressful life events from T1 to T2. An increase in reported stressful life events predicted an increase in PTSS (β =1.481, 95% CI .552 to 2.411). Length of stay, increase in stressful life events and level of education did not predict changes in the HSCL-37A.

**Conclusions:**

There was no average change in the level of PTSS, depression, anxiety, or externalizing problems in this group of unaccompanied refugee children from shortly after arrival to nearly two years later. The large variation in change scores across informants indicates a need for monitoring the development of mental health problems and securing that the youth’s primary psychosocial needs are met. The high rate of children above clinical cut-off on the symptoms scales and with suicidal ideation indicates that many may be in need of treatment.

## Background

A large body of research has documented that refugee children develop symptoms of post-traumatic stress disorder (PTSD) and other mental health problems at high rates [1, 2]. The reported symptoms include anxiety problems, depressive symptoms, and behavioral problems [3, 4]. These symptoms are often linked to trauma exposure prior to migration [5–8] and the loss of the primary caregiver [9–11].

Due to a lack of longitudinal studies, little is known concerning how refugee children and adolescents’ mental health problems change over time as they resettle in a host country [4]. There are particularly few studies on the youngest unaccompanied children who arrive to a host country when under the age of 15. On the one hand, we may assume that leaving areas of ongoing conflict or war-afflicted countries and resettling in countries where one is safe from war-related atrocities may lead to an alleviation of mental health problems. On the other hand, the transition from one country to a second country involves changes in many aspects of daily life, including new school settings, different foods, religious and cultural traditions, and potential experiences of discrimination and isolation. This process of cultural transition has been defined as acculturation, and the associated stress has been labeled as acculturative stress [12]. Many refugee children and adolescents experience such acculturative stress [13] and stress that is associated with migration and displacement as well [4]. Thus, children who escape war and violence may be at a high risk for developing mental health problems because the acculturation and adjustment processes are superimposed on the prior exposure to trauma and its consequences.

Refugee children who flee from their home country without parental figures are considered to be particularly vulnerable [14–16]. In one study in which unaccompanied and accompanied minors’ mental health was compared unaccompanied refugee minors consistently reported significantly higher scores for internalizing problems, traumatic stress reactions, and stressful life events than accompanied minors [9]. Several studies have shown that parental support is essential in reducing the risk for developing mental health problems following trauma [1, 17]. Children are dependent on adults for protection, care, and decision-making [9, 18]. Therefore, the process of acculturation may be particularly stressful for these children because they do not have parental figures to provide guidance and support and they lack parental protection from new stressors and/or traumas. Also it can be difficult for these children to form new attachment figures, new friendships and build a supporting network in the host country. They are often placed in centers with few possibilities to form new relationships and many experience several relocations with new ruptures in relations [19].

In sum, the literature has suggested that stressors due to 1) trauma and loss and 2) acculturation and resettlement impact immigrant refugee children who are in resettlement. However, not all unaccompanied refugee children report having clinically significant symptoms of mental health problems [1, 4, 20, 21]. Unfortunately, few longitudinal studies have described the patterns and pathways of recovery for these children [4, 22], and there are significant gaps in the literature regarding the various trajectories of such problems during resettlement and the predictors of changes in mental health [1, 4, 16].

One factor that may influence mental health development post-resettlement for unaccompanied minors is the time since arrival in the new host country [4]. Although some studies have indicated that time to adjust to the host country can improve functioning [20], the research findings have been mixed [5, 16]. Some cross-sectional studies have shown that the amount of time that unaccompanied young refugees live in their host country does not impact symptom decline [23–25]. These findings are supported by two longitudinal studies of unaccompanied minors that were conducted in the Netherlands [5] and Belgium [16]. Both these studies indicate that mental health problems tend to persist and become chronic.

Lastly, studies have shown that amount of time in the host country is negatively associated with depression but not with post-traumatic stress symptoms [26]. This result has led scholars to believe that depression and post-traumatic stress symptoms may have distinct predictors and that they should be examined separately. Depressive symptoms may be more affected by current stressful events and loss, and PTS symptoms may have a greater association with recent and prior trauma [8]. For instance, the feeling of sadness over the loss of friends and family and acculturation difficulties may initially be counterbalanced by hopes for a safer and more prosperous life. Over time, this hope may be replaced by despair and shattered expectations.

In addition to time, a second factor that may influence mental health trajectories is stressful life events, particularly trauma exposure. To date, trauma exposure is the most consistent predictor of long-term mental health problems among this group of children [4, 5, 16]. A recent review indicated that 16% of children and adolescents who were exposed to trauma developed PTSD [27]. Interpersonal traumas that are chronic in nature or that affect social support systems and core assumptions about the world being safe and benevolent, as occurs with many war-related experiences, tend to lead to higher rates of PTSD and poorer daily functioning than single incident traumas [27]. Importantly, unaccompanied refugee children may continue to experience traumatizing situations or other stressful life experiences after resettlement [4]. Thus, they are vulnerable to continuous mental health problems. Therefore, an examination of whether changes in the reporting of traumatic and stressful experiences are associated with changes in mental health problems may clarify why different patterns of recovery are reported in the current literature.

It is also important to examine protective factors, and one potential protective factor is length of education [1]. The experience of some formal schooling may be indicative of reading and writing competencies that may make adjustment easier. However, few studies have investigated the relationship between level of education in the homeland and later adjustment [4]. One exception is a study in the Netherlands, which did not find a relationship between level of education and later mental health problems in a group of refugee minors [5]. The potential role of education as a predictor of refugees’ long-term mental health or mental health development is unclear and remains important to investigate [4].

In the current paper, we report on the development in PTSS and general mental health symptoms in a sample of unaccompanied refugee children from shortly after their arrival to Norway (T1) to approximately two years post-resettlement (T2). At arrival, the participant’s self-reported age was below 15 years of age. The first research question is how PTSS and general mental health symptoms changed from T1 to T2. Since longitudinal studies, particularly those that examine the youngest group of children, are virtually non-existent, it is difficult to make clear predictions. In Norway asylum-seeking children who are under the age of 15 and arrive without legal guardians are placed in the care of the child protection services. They are placed in small units of care, and professionally trained adults care for them. They attend the local school, and their basic psychosocial needs are addressed. Asylum-seeking children under the age of 16 arriving into Norway without legal guardians are normally granted a three years permit to stay in the country. After the three years, as a rule, they are given a permanent permit to stay. Exceptions to this rule can be made if they are convicted of a serious crime or they have given wrong identity or other types of erroneous information to the authorities. Then they may be sent out of the country, even if they have obtained a permanent permission to stay. If they are granted a permit to stay in the country the youth are then transferred to a municipality where they for the most part live in a home with other refugee minors and professional staff or are placed into a foster family. Some may have other types of living arrangements but this is unusual.

Since the children in Norway are place into smaller units of care where their basic psychosocial needs are addressed, and where they, for the most part, are granted permanent residency so they do not have to fear being sent home against their will, we could expect a decline in mental health problems. On the other hand prolonged sadness and grief over losses in the home country, worry about family members, unresolved traumatic experiences, loss of attachment figures and social support, in addition to struggles with acculturation may maintain mental health problems [1, 20, 28]. The second research question is whether there are gender differences in symptom development over time. Research has found that females are at greater risk for developing mental health problems, particularly PTSD, after trauma [29]. However, a review of psychiatric symptoms among young refugees showed that gender does not consistently emerge as either a risk or protective factor and that more studies are needed [4].

The final research question is whether length of stay, change in number of stressful life events from T1 to T2, and length of education predict changes in post-traumatic stress symptoms (PTSS) and general mental health symptoms. We hypothesize that greater length of stay and education are associated with fewer PTSS and general mental health problems and that an increase in reported traumatic and stressful experiences predicts increases in PTSS and general mental health problems.

## Method

### Procedure and participants

In this follow-up study, we reassessed unaccompanied refugee minors who had participated in a study (n =93) approximately 6 months after arriving in Norway and while they were living in small care units run by the State Child Protection Services. (For a description of the sample and recruitment procedures, see the authors’ publication – [7]). The follow-up (T2) took place 1.9 years, on average, after the first assessment (SD =0.6, range 0.9 to 2.8 years) and comprised the same measurements in addition to a semi-structured interview. The follow-up assessments were administered over a one-year period (fall 2012 to fall 2013).

The sample at T2 included 75 participants, with a majority of boys (n =62; 83%). The participants’ reported mean age at T2 was 16.5 years (SD =1.6, range 13.5 to 20.7 years).^a^ The children originated from 12 different countries. The most common countries of origin were Afghanistan (n = 38; 50.7%), Eritrea (n = 12; 16.0%), Somalia (n = 9; 12.0%), and Sri Lanka (n = 6; 8.0%). At the time of assessment, all but three of the children were resettled in the municipalities. Forty-eight children (64.0%) lived with two or more other refugee minors in a house that was run by the council or private organizations and staffed with personnel 24 hours per day. Seven children lived in small one-room apartments, 10 lived in foster families, 3 in asylum reception centers, and 7 had other types of living arrangements. In terms of education, 41.3% of the children had more than 3 years of schooling, 30.7% had some but less than 3 years of schooling, and 14.7% had no schooling. The educational level data were missing for 13.3% of the participants. Nine of the participants from T1 (1 female) were out of the country or missing at the time of the follow-up. Furthermore, 11 participants did not participate because either the child (9) or the legal guardian (2) did not provide informed consent. No systematic information on reasons for non-participation is available. However, there were no significant differences in any of the T1 symptom total or subscales or the number of stressful life events between those who participated at T1 and those who were lost to follow-up.

The children were contacted either directly by the researcher or by their legal guardian and provided verbal informed consent. The participants completed the questionnaires on a computer, where the questions were presented either in their native language or Norwegian both verbally and written. For nine cases, the questionnaires were not translated into the native language and the participants were not able to understand Norwegian; therefore, an interpreter was present. The questionnaires were administered by a clinical psychologist. The study was approved by The National Committee for Medical and Health Research Ethics.

### Measures

#### Hopkins symptom checklist-37 A for adolescents

The Hopkins Symptom Checklist-37 A for Adolescents (HSCL) [30] is a self-report measure that was developed for unaccompanied asylum-seeking children. It includes 10 anxiety items (e.g., *Feeling tense or keyed up*), 15 depression items (e.g., *Crying easily*), which together create a 25-item internalizing subscale, and 12 externalizing items (e.g., *Arguing often*). The items are rated on a 4-point scale that ranges from 1 (*never*) to 4 (*always*). In the current study, four HSCL items were omitted, as these items were deemed inappropriate due to the participants’ young age at the first assessment. These items were three externalizing questions (*Drinking alcohol when I go out on the weekend (4); Drinking alcohol during the week (25); Using drugs (37*)) and one item from the depression subscale (*Loss of sexual interest (13)*). The conservative value 1 (*never*) was inserted for these four items. In the current study (T2), the inter-item reliability of the total HSCL (37 items, α = .94) and the HSCL internalizing subscales (anxiety, α = .90; depression α = .91; the total internalizing scale α = .95) was good. The HSCL externalizing scale reliability was satisfactory (12 items, α = .64). The suggested clinical cut-off scores are as follows: total score = 69.0, anxiety = 20.0, and depression = 33.2. No cut-off has been suggested for the externalizing scale [23]. *Stressful life events.* The Stressful Life Events (SLE) measure [31] is a checklist of 12 dichotomous (*yes/no*) questions about the experience of severe life events. The SLE covers three primary areas of events (i.e., *separation from family, physical or sexual violence,* and *war or armed conflict*).

For the HSCL and the SLE, we used the translations that were offered by the Centrum ‘45 in the Netherlands. This center also validated the instruments and translations [32].

#### Child PTSD symptom scale

The Child PTSD Symptom Scale (CPSS) [33] is a 17-item self-report questionnaire that was developed for children and youth between 10 and 18 years of age. It examines the PTSD symptoms that are described in the DSM-IV manual. The child rates the symptom frequency for the previous 2 weeks using a 4-point scale that ranges from 0 (*Not at all*) to 3 (*5 or more times a week/almost always*). The CPSS comprises three subscales, re-experience (five items, e.g., *having bad dreams or nightmares*), avoidance (seven items, e.g., *Tried not to think, talk or feel about the event(s)*) and hyperarousal (five items, e.g., *Trouble falling asleep or sleeping through the night*). The CPSS has demonstrated convergent validity, internal consistency, and test–retest reliability [33]. In the current study, the inter-item reliability for the CPSS total scale (17 items, α = .91) and the subscales (re-experience, α = .88; avoidance, α = .78; hyperarousal, α = .72) was good. The suggested cut-off score for the CPSS is a total score of 11 or higher [33].

The CPSS was translated and back translated using recommended translation procedures. The diagnostic utility of the CPSS has been studied in a clinical sample of traumatized children in Norway showing it to be a good tool for screening purposes [34].

### Statistical analysis

All of the analyses were performed with IBM Statistics SPSS, version 21.0. The data analyses progressed through the following main steps. First, descriptive statistics were run to examine the scores at T2 and the change scores from T1 to T2. Second, Pearson’s r-correlations were calculated for all variables between time points. We used the following criteria to determine the correlation sizes: <.10 = low, .10-.29 = small, .30-.49 = moderate, and > .50 = high [35]. Third, independent samples t-tests were run to examine gender differences in the change scores. The effect size differences were calculated by subtracting the girls’ mean scores from the boys’ mean scores divided by the pooled standard deviation between the groups. We used the following criteria to determine the effect sizes: >.10 = small, >.30 = medium, >.50 = large [35]. Finally, the predictor variables (length of stay, change in stressful life events from T1 to T2, and education) were entered in a multiple linear regression model for all symptom scales (HSCL total and subscales; CPSS total and subscales). Education was calculated as a continuous variable with three levels (No education =1; Three years or less of education =2; More than three years of education =3).

## Results

On average, there was little change in the mean scores of the symptom scales between T1 and T2. None of the change scores were significant. There was, however, a significant increase in reported stressful life events from T1 to T2 (M change =0.8, SD =2.6, 95% CI for the difference 0.2 to 1.4, t =2.586, p < .05).

**Figure 1:**
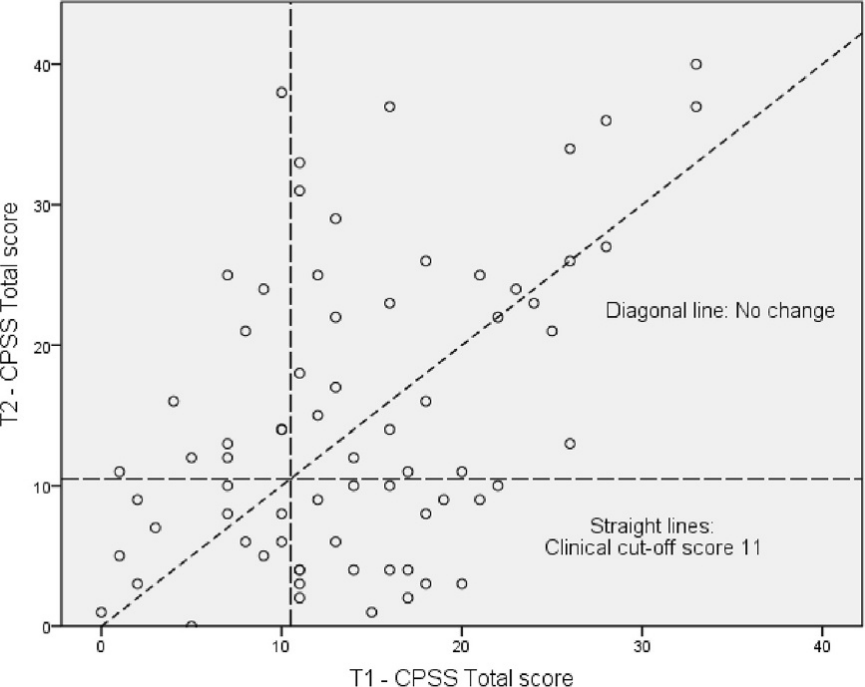
**Changes in CPSS scores from T1 - T2** ^**1**^ **.** ^1^ Each circle represents an unaccompanied minor. The diagonal line indicates “No change”; thus, the individuals who are above this line showed more symptoms at T2 than at T1 and vice versa. The upper-right square shows the individuals whose scores on the actual scale were above the clinical cut-off both at T1 and T2 (constantly high level of symptoms). The upper-left square shows those whose scores were below the cut-off at T1 and above the cut-off at T2 (deteriorated). The lower-left square shows those whose scores were below the cut-off at both T1 and T2, and the lower-right square shows those whose scores were over the cut-off at T1 and below at T2 (improved).

**Figure 2:**
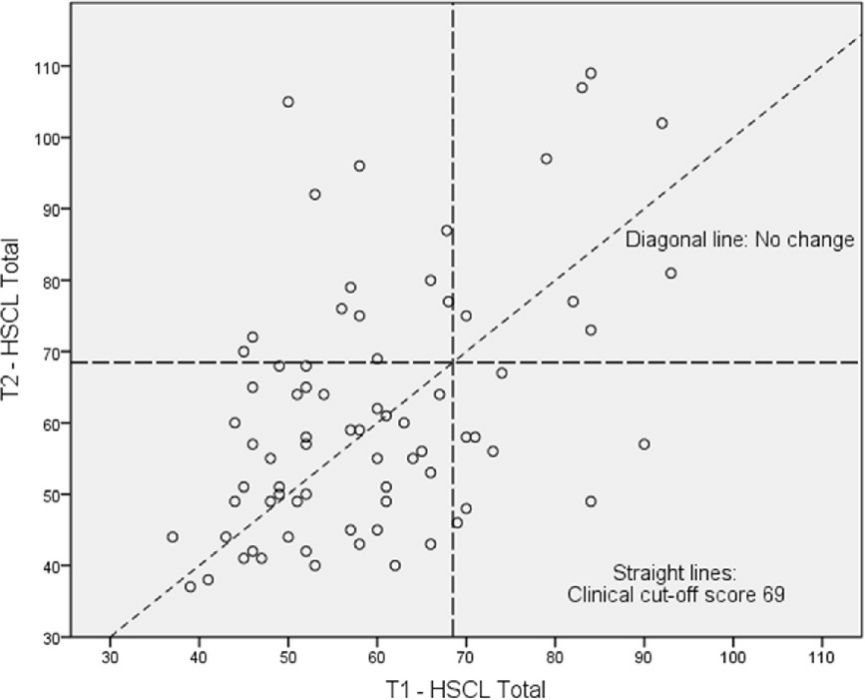
**Changes in HSCL – 37 A scores from T1 - T2** ^**2**^ **.** ^2^ Each circle represents an unaccompanied minor. The diagonal line indicates “No change”; thus, the individuals who are above this line showed more symptoms at T2 than at T1 and vice versa. The upper-right square shows the individuals whose scores on the actual scale were above the clinical cut-off both at T1 and T2 (constantly high level of symptoms). The upper-left square shows those whose scores were below the cut-off at T1 and above the cut-off at T2 (deteriorated). The lower-left square shows those whose scores were below the cut-off at both T1 and T2, and the lower-right square shows those whose scores were over the cut-off at T1 and below at T2 (improved). Note that for the HSCL scale, the cut-off score of 69 is not adjusted, as 4 items are given the default value of 1. Such an adjustment would have resulted in a cut-off value of 62 and approximately 10 additional individuals above the cut-off value for both of the points in time.

Table 1 provides an overview of the T1, T2, and change scores. The change scores for the different symptom scales were significantly correlated, and these correlations were large. See Table 2 for details. Although, at the group level, there was no difference in either PTSD symptoms or general mental health symptoms between T1 and T2, the large standard deviations of both of the change variables indicated considerable individual variation in the degree of improvement versus decline. The scatterplots in Figures 1 and 2 illustrate the spread in change scores between informants. Of the 47 participants who scored above the CPSS total scale clinical cut-off value at T1, 28 (59.6%) remained above the cut-off at T2, and 19 (40.4%) decreased to below the cut-off. Of the 23 participants who scored below the CPSS total scale clinical cut-off value at T1, 12 (52.2%) remained below the cut-off and 11 (47.8%) increased to above the cut-off at T2. The information of five participants was missing because the CPSS was not administered to them at T1.

**Table 1:** **Symptom measures of 75 unaccompanied refugee children at two time points**

Scale	T1	T2	Change from T1 to T 2 ^1^	Corr. btw time points
	M (SD)	M (SD)	M (SD)	
HSCL-Tot	59.4 (13.3)	61.4 (17.5)	2.0 (16.4)	.47**
HSCL-Ext	14.6 (2.6)	14.9 (2.84)	0.3 (3.3)	.28*
HSCL-Int	44.3 (12.5)	46.4 (15.6)	2.1 (14.4)	.49**
HSCL-Anx	17.3 (4.7)	18.0 (6.5)	0.7 (5.8)	.51**
HSCL-Dep	27.1 (8.3)	28.5 (9.6)	1.4 (9.7)	.42**
CPSS-Tot	14.1 (7.6)	14.9 (10.8)	0.8 (9.9)	.47**
CPSS-Rexp	5.0 (3.1)	4.6 (3.9)	-0.4 (4.5)	.20*
CPSS-Hyper	4.1 (2.9)	4.5 (3.5)	0.5 (3.4)	.46**
CPSS-Avoid	5.1 (3.5)	5.8 (4.6)	0.8 (4.9)	.31*
SLE	5.8 (2.2)	6.6 (2.1)	0.8 (2.6)*	.29*

*Note*. Corr = Pearson’s r-correlations. HSCL = Hopkins Symptom Checklist 37A. Tot = Total. Ext = Externalizing scale. Int = Internalizing scale. Anx = Anxiety scale. Dep = Depression scale. CPSS = Child PTSD Symptom Scale. Rexp = Re-experience subscale. Hyper = Hyperarousal subscale. Avoid = Avoidance subscale. SLE = Stressful Life Events measure. ^1^Negative scores indicate improvement. *p < .05, **p < .001.

**Table 2:** **Correlations between change scores for 75 unaccompanied refugee children**

Scale	HSCL-Ext	HSCL-Int	HSCL-Anx	HSCL-Dep	CPSS-Tot	CPSS-Re-exp	CPSS-Hyper	CPSS-Avoid	SLE
HSCL-Tot	.73**	.99**	.83**	.97**	.81**	.76**	.69**	.67**	.41**
HSCL-Ext		.65**	.55**	.65**	.62**	.59**	.53**	.51**	.31**
HSCL-Int			.91**	.97**	.77**	.75**	.64**	.67**	.42**
HSCL-Anx				.79**	.73**	.72**	.63**	.61**	.43**
HSCL-Dep					.75**	.71**	.62**	.65**	.38**
CPSS-Tot						.84**	.77**	.88**	.38**
CPSS-Rexp							.49**	.67**	.40**
CPSS-Hyper								.60**	.25*
CPSS-Avoid									.31*

*Note*. Corr = Pearson’s r-correlations. HSCL = Hopkins Symptom Checklist-37A. Tot = Total. Ext = Externalizing scale. Int = Internalizing scale. Anx = Anxiety scale. Dep = Depression scale. CPSS = Child PTSD Symptom Scale. Reexp = Re-experience subscale. Hyper = Hyperarousal subscale. Avoid = Avoidance subscale. SLE = Stressful Life Events measure. *p < .05, **p < .001.

Of the 16 participants who scored above the HSCL total scale clinical cut-off value at T1, eight (50.0%) remained above the cut-off at T2, and eight (50.0%) decreased to below the cut-off. Of the 57 participants who scored below the HSCL total scale clinical cut-off value at T1, 45 (78.9%) remained below the cut-off at T2 and 12 (21.1%) increased to above the cut-off at T2. The information of two participants was missing due to a lack of T1 data.

Alarmingly, there was a significant mean increase in the single item “suicidal ideation” of the HSCL scale between T1 and T2, and 8 participants (10.7%) rated the two top values on this item at T2 (i.e., often or always having “thoughts of ending my life” in the last month).

We examined whether there were significant gender differences in the HSCL and CPSS scales at T2 or in the change scores between T1 and T2. The results are displayed in Table 3. The independent samples t-tests indicated no significant gender differences in the HSCL total scale or any of the HSCL subscales. Similarly, there were no significant differences in the CPSS total scale or any of the CPSS subscales. The CPSS hyperarousal subscale approached significance (*p* = .098), with girls demonstrating greater deterioration than boys. Further examination indicated small to medium effect size differences on the HSCL scales and small effect size differences on the CPSS scales, with the exception of the hyperarousal subscale, which evidenced a large effect size difference. See Table 3 for details.

**Table 3:** **Gender differences in the CPSS and HSCL scores of 75 unaccompanied young asylum seekers**

Scale	M change boys (SD)	M change girls (SD)	***t***-value	95% CI of difference	***p***-value	Effect size ***d***
HSCL Tot	1.13 (16.89)	6.33 (13.75)	-1.001	-15.55 to 5.15	.320	-0.31
HSCL Int	1.47 (14.98)	5.58 (10.90)	-.904	-2.71 to 1.50	.379	-0.28
HSCL Ext	0.15 (3.26)	0.75 (3.79)	-.571	-13.19 to 4.96	.570	-0.18
CPSS Tot	0.72 (9.78)	1.25 (10.76)	-.167	-6.82 to 5.77	.868	-0.05
CPSS Rexp	-0.24 (4.49)	-1.08 (4.46)	.592	-1.99 to 3.68	.556	0.04
CPSS Hyper	0.16 (3.40)	1.92 (2.81)	-1.679	-3.86 to 0.33	.098	-0.53
CPSS Avoid	0.82 (4.52)	0.42 (6.50)	.262	-2.70 to 3.51	.794	0.01

*Note*. HSCL = Hopkins Symptom Checklist 37A. Tot = Total. Ext = Externalizing scale. Int = Internalizing scale. Anx = Anxiety scale. Dep = Depression scale. CPSS = Child PTSD Symptom Scale. Reexp = Re-experience subscale. Hyper = Hyperarousal subscale. Avoid = Avoidance subscale.

Next, we examined whether length of stay, change in SLE, or length of education predicted change from T1 to T2. The multiple regression model was first run for the HSCL total scale. The model was non-significant (*adj. r*^*2*^ = .03, *p* = .176), as were all of the predictor variables (*p* > .068). The models were also non-significant for the HSCL externalizing subscale (*adj. r*^*2*^ = -.01, *p* = .494), the HSCL internalizing subscale (*adj. r*^*2*^ = .04, *p* = .130) and the two subscales of the internalizing subscale, HSCL anxiety (*adj. r*^*2*^ = .07, *p* = .052) and HSCL depression (*adj. r*^*2*^ = -.01, *p* = .316).

Subsequently, we ran multiple regression models using the same predictor variables on the CPSS total scale. The model was significant for total CPSS (*adj. r*^*2*^ = .11, *p* < .05) and explained 11% of the variance in change in CPSS total symptoms. Change in SLE was the only significant single predictor in the model (*p* < .05, β =1.481, 95% CI .552 to 2.411). In terms of the CPSS subscales, the model was significant both for the CPSS re-experience subscale (*adj. r*^*2*^ = .10, *p* < .05), with change in SLE as the only significant predictor (*p* < .05, β = .667, 95% CI .245 to 1.089), and for CPSS avoidance subscale (*adj. r*^*2*^ = .08, *p* = .043), still with change in SLE as the only significant predictor (*p* < .05, β = .602, 95% CI .141 to 1.064). The model was non-significant for the CPSS hyperarousal subscale (*adj. r*^*2*^ = -.02, *p* = .666).

Age was not included in the regression models due to the variable’s uncertain validity. However, as a post-hoc analysis, all of the age variables (T1 and T2) were included in the regression models. The model that predicted HSCL total change remained non-significant after the inclusion of the age variables. For the model that predicted CPSS total change, the age variables were non-significant predictors, but the overall model remained significant (details are available upon request).

In summary, on average, there were no significant changes in symptoms from T1 to T2, although there was considerable variation between the participants. Length of stay, change in number of stressful life events, and level of education did not predict changes in anxiety, depression or externalizing symptoms. The model that predicted change in PTSD symptoms was significant, explaining 11% of the variance. Change in reported stressful life events was the only significant predictor. In terms of the CPSS subscales, the model significantly predicted both the re-experience and the avoidance subscales; it did not predict the hyperarousal subscale.

## Discussion

This two-year follow-up assessment of unaccompanied refugee children showed that symptoms of PTSD, depression, anxiety and externalizing problems were on average unchanged from an earlier assessment that was conducted 6 months after arrival in Norway. This finding is consistent with the findings from Bean and colleagues in the Netherlands [5] and Vervliet and colleagues in Belgium [16] and indicates a chronic course of stress reactions and that mental health problems do not change significantly over time. This may not be surprising since these youth have experienced multiple traumas in their homeland and they have fled their home country without caregiver’s protection and care. Particularly since this study encompasses very young asylum seekers the impact of being without attachment figures may be detrimental to their mental health and contribute to the maintenance of PTSS and other symptoms. It is for instance alarming that 11% of the participants reported high scores on suicidal ideation. This result indicates a need for health workers to pay specific attention to suicidal ideation in this group.

However, it is also notable that many children showed signs of recovery despite their serious traumatization and numerous losses. It may be that for these youth being in a safe environment away from war related traumas has led to less stress and mental health problems. Their living arrangements have been small units of care where their basic psychosocial needs are taken care of and this may have aided their recovery process. The large variation in the development of mental health problems is important and indicates the need for monitoring health problems over time and providing treatment for those with impaired development.

Our study does not provide answers as to why so many youth experience continuing or elevated problems. Previous studies have mentioned uncertainty in asylum status as a stressor, but as a rule in Norway all unaccompanied asylum seekers are granted permanent residency. Also type of residency has been mentioned as influencing mental health with youth living in foster care fare better than youth in other living arrangements [36]. Unfortunately, due to the small sample size we did not have power to examine living arrangements as an independent variable in our study. Future studies should examine this closer. From developmental psychology and the trauma literature it is reasonable to assume that the young age of the participants in this study and the fact that they arrive in Norway without caregivers has a negative impact on their development. Coping with a new environment, a different culture, and a foreign language is considered stressful for all asylum-seekers. Being without attachment figures for guidance, protection and care adds to this burden. In addition many worry about family member’s wellbeing at home and many experience new losses while in exile. All these conditions may maintain or lead to elevated anxiety and depression and also contribute to the maintenance of posttraumatic stress symptoms. Research should examine all these factors more closely to disentangle the different pathways described in this study and that also have been found in refugee youth with caregivers [37].

Previous longitudinal studies have found a separation between the long-term sequelae of PTSD and other mental health problems [8]. Similar to Bean et al. [5], in the current study, the change patterns between the different symptom scales were similar and the correlations between the scales were high, so more research is needed to support this claim.

The only significant predictor of symptom change that was identified in the current study was change in the reporting of stressful life events. This change predicted change in PTSS only; it did not predict change in general symptoms. It may be that some of these children may have experienced new adversities such as new family losses that may have contributed to the maintenance of PTSS. It is important to acknowledge that children’s inconsistency in the recall of emotionally upsetting events is not uncommon and has been reported in samples of non-refugee children [38] and in refugee samples [16, 39]. These inconsistencies may have different explanations that are related to memory loss or mental health problems such as post-trauma avoidance reactions. Over time, some children may feel comfortable revealing traumas that they previously omitted [39]. They may also have misreported life experiences due to fear of the asylum-seeking process. We found that the re-experiencing symptoms were particularly elevated among the children who reported more stressful life experiences at T2 then T1. This result could be an indication that some children are “allowing” themselves to think about and remember past events.

Length of stay and education level did not predict change in PTSD symptoms or general mental health problems. Despite the lack of previous studies, we had anticipated that the experience of some schooling (possibly indicating reading and writing capacity) would aid children in adjusting to the new country and ease, for instance, their integration into school through academic achievements. Although this relationship may exist, is does not seem to influence the children’s mental health trajectories. Unfortunately, we do not have systematic information on the children’s academic achievement.

We have not been able to study other variables such as worries about family back home, feelings of pressure to fare well in the host country and contribute money to relatives back home or other daily stressors these youth may have experienced. In a longitudinal study on unaccompanied minors in Belgium, Vervliet and colleagues [16] found that the mean number of reported daily stressors increased over time. Commonly reported stressors were discrimination, dissatisfaction with education situation, being forcibly moved and missing family. It may be that many youth in our study have these types of experiences and that these contribute to the maintenance of symptoms. Additional studies are needed to examine this further. Also it may be that the follow-up period of two years in this study is too short to expect changes in mental health symptoms. At least one study on unaccompanied minors has found that changes occur at a later stage [40] and more studies are needed to confirm this.

### Strengths and limitations

The current study has several strengths. First, it is one of few follow-up studies on young unaccompanied refugee children, and perhaps the only study of the youngest group of refugees. Second, the attrition rate from T1 to T2 was quite low. Third, the measures that were used have frequently been used with refugee children; therefore, comparisons with other studies are feasible. Finally, clinical psychologists administered the assessments and provided assistance if needed, and interpreters were available for those who needed additional translation.

Despite these strengths, the current results must be interpreted in light of certain limitations. The follow-up measures were administered approximately two years after the youth arrived to Norway. Since studies have shown that the first years in exile represent special challenges regarding for instance acculturation, uncertainty as to asylum status, and sadness over loss of friends and broken attachment bonds to family, further assessments at later time periods would have been valuable.

Also, mental health problems were assessed using a checklist rather than a diagnostic interview; therefore, diagnostic inferences cannot be made. Simple checklists may fail to capture the wide range of problems that these children experienced. Although it is out of the scope of this paper to discuss whether instruments developed to assess psychological problems in Western populations are valid for non-Western populations [41, 42], our study’s results should be understood in light of this limitation. In addition, we examined a limited number of predictors, and important issues such as immigration status, experiences with daily stressors, social support, and the use of mental health services were not systematically assessed. Although the girls had a tendency to report more increased symptoms than the boys on some scales, the sample size was small and the gender groups were unbalanced. Therefore, the role of gender in symptom development should be examined in future studies with greater power. Although the gender balance in this study reflects the ratio of asylum-seekers in Norway, where most are male, a greater number of girls in the follow-up may have enabled us to detect significant gender differences. Future studies should examine age differences because the findings thus far seem to be inconclusive [4]. We were also unable to examine potential differences in living situation for the development of symptoms, and this should be examined in future studies.

## Conclusions

The refugee children in this study show different patterns of mental health development. Alarmingly, many of the children showed no improvement or deterioration. The stably high levels of post-trauma reactions, anxiety, and depression that were shown in this group of young refugees reveal these children’s vulnerability and indicate a chronic course. We must bear in mind that this study examined the mental health trajectories of rather young children who fled from their homeland without the protection of caring adults. These children may be the most vulnerable refugees.

Additional studies are needed to understand the risk and resilience factors for mental health problems in refugee children who are particularly vulnerable due to the absence of parents for protection and care. Due to the lack of longitudinal studies, it is difficult to identify children who are at risk, and such studies are needed, particularly for the youngest refugee children. The current study indicates that focus should be put into how the youth are received at arrival in their host country and how they are given possibilities to make new relationships and build systems of social support. It is important that their basic needs for care are met. Because some children seem to show patterns of improvement but many do not, systems of continuing assessments and monitoring adjustment should be implemented. This should include the children’s own perspectives of what needs they have. Furthermore, treatment should be provided to children with elevated symptoms.

Studying and comparing research on refugee minors poses several challenges. For one, refugees come from different countries of origin, their sociocultural background is varied, and their developmental history, including the stability in attachment figures, is often unknown. There are also variations in reasons for leaving their home country and whether their caregivers are alive or not. Some may have come to a new country for the purpose of making a good living and to provide support to family members in their home country. Many also worry about how their family is doing in their home country. These conditions may put undue pressure on the youth and add to their daily burdens in the host countries. Also it is difficult to compare longitudinal studies from different countries because countries have diverse practices regarding how unaccompanied refugee children are taken care of in the host country. In some countries they are placed in large reception centers while other countries place them in smaller units of care or in foster homes. Also countries have different legislature and practices for who is granted asylum. Fear for being sent back to their country of origin may add to life stress, worry, and mental health problems. All these factors may contribute to differences in mental health trajectories.

## Endnote

^a^Note that between the T1 and T2 assessments, age was corrected for 25 of the participants due to age assessments from the Norwegian Directorate of Immigration. Of these participants, 5 were given a younger age and 20 were given an older age. For one male 4 ½ years was added to his age thus accounting for the large range in age at T2.

## Abbreviations

PTSD: Post-traumatic stress disorder
PTSS: Post-traumatic stress symptoms
CPSS: Child PTSD symptom scale
HSCL: The Hopkins symptom checklist-37 A for adolescents
SLE: Stressful life events.
